# In Vitro Effect of the Cell-Free Supernatant of the *Lactobacillus casei* Strain IMAU60214 against the Different Pathogenic Properties of Diarrheagenic *Escherichia coli*

**DOI:** 10.3390/microorganisms11051324

**Published:** 2023-05-18

**Authors:** Luz María Rocha-Ramírez, Ulises Hernández-Chiñas, Silvia Selene Moreno-Guerrero, Arturo Ramírez-Pacheco, Carlos A. Eslava

**Affiliations:** 1 Unidad de Investigación en Enfermedades Infecciosas, Hospital Infantil de México Federico Gómez, Dr. Márquez No. 162, Col. Doctores, Alcaldía Cuauhtémoc, Ciudad de México 06720, Mexico; 2 Unidad Periférica de Investigación Básica y Clínica en Enfermedades Infecciosas, Universidad Nacional Autónoma de México, Dr. Márquez 162, Col. Doctores, Alcaldía Cuauhtémoc, Ciudad de México 06720, Mexico; ulisesh@unam.mx (U.H.-C.); eslava@unam.mx (C.A.E.); 3 Departamento de Salud Pública, División de Investigación, Facultad de Medicina, Universidad Nacional Autónoma de México, Ciudad de México 04510, Mexico; 4 Departamento de Hemato-Oncología, Hospital Infantil de México Federico Gómez. Dr. Márquez No. 162, Col. Doctores, Alcaldía Cuauhtémoc, Ciudad de México 06720, Mexico; sswitch@yahoo.com (S.S.M.-G.); artur_tauro@yahoo.com.mx (A.R.-P.)

**Keywords:** *Lactobacillus casei* IMAU60214, cell-free supernatant, enterohemorrhagic *Escherihcia coli* enteroaggregative, *E. coli*, anti-biofilms, coaggregation, anti-cytotoxins, antimicrobial activity

## Abstract

Enteroaggregative *Escherichia coli* (EAEC) and enterohemorrhagic *E. coli* (EHEC) are *E. coli* pathotypes associated with unmanageable diarrhea in children and adults. An alternative to the treatment of infections caused by these microorganisms is the use of the bacteria of the *Lactobacillus* genus; however, the beneficial effects on the intestinal mucosa are specific to the strain and species. The interest of this study consisted of analyzing the coaggregation properties of *Lactobacillus casei* IMAU60214, as well as the effect of cell-free supernatant (CSF) on growth and anti-cytotoxic activity in a cell model of the human intestinal epithelium for an agar diffusion assay (HT-29) and the inhibition of biofilm formation on plates of DEC strains of the EAEC and EHEC pathotypes. The results showed that *L. casei* IMAU60214 exhibits time-dependent coaggregation (35–40%) against EAEC and EHEC that is similar to the control *E. coli* ATCC 25922. The CSF showed antimicrobial activity (20–80%) against EAEC and EHEC depending on the concentration. In addition, the formation and dispersion of biofilms of the same strains decrease, and the proteolytic pre-treatment with catalase and/or proteinase K (1 mg/mL) of CSF reduces the antimicrobial effect. When evaluating the effect in HT-29 cells pre-treated with CFS on the toxic activity induced by the EAEC and EHEC strains, a reduction of between 30 and 40% was observed. The results show that *L. casei* IMAU60214 and its CSF have properties that interfere with some properties associated with the virulence of the EAEC and EHEC strains that cause intestinal infection, which supports their use for the control and prevention of infections caused by these bacteria.

## 1. Introduction

*Escherichia coli* is a Gram-negative bacillus that colonizes the intestinal mucosa of humans and other animal species at birth. The bacterium integrates and forms part of the microbiota of its host by establishing a mutualistic relationship, which is why the bacteria have been defined as *E. coli* [1]. However, the plasticity of its genome together with the horizontal transfer of genetic information has favored the emergence of clones of the bacterium responsible for different clinical syndromes such as diarrhea, urinary tract infections, sepsis, and meningitis [2,3]. Diarrheagenic *E. coli* (DEC) strains represent the second leading cause of infectious intestinal diseases in children and young adults, second only to enteric infections caused by viral pathogens such as rotavirus, calicivirus, adenovirus, and norovirus [4,5,6]. DEC strains present different antigenic characteristics (serogroups and serotypes) and virulence properties that cause various clinical features. That is why the term “pathotypes” or pathogenic groups arose, in which their properties are considered, including the type of adherence related to the type of adhesins they express, the production of toxins with enterotoxic or cytotoxic effects, or the ability to invade cells [6,7]. Pathotypes include enteropathogenic *E. coli* (EPEC), enterohaemorrhagic *E. coli* (EHEC), Shiga-toxin-producing *E. coli* (STEC), Enteroaggregative *E. coli* (EAEC), enterotoxigenic *E. coli* (ETEC), enteroinvasive *E. coli* (EIEC), and hybrid strains with more than one virulence factor, such as enteroaggregative hemorrhagic *E. coli* (EAEHEC) [8,9]. In addition to the pathotypes of DEC strains, strains that cause extraintestinal infections (EXPEC) causing urinary tract infections (UPEC), meningitis (NMEC), or septicemia have been described. Another relevant aspect of infectious diseases is related to the increase in resistance to antimicrobials, a situation that is increasing every day, which is why the World Health Organization (WHO) has pointed out the possibility that, by the year 2050, there could be no useful treatments for infectious diseases [10]. Given this possibility, it raises the need to seek alternatives for the management of infections, including those related to *E. coli*, which has been defined as priority number one within the community and in intrahospital infections [11]. An alternative that has been used for a long time is bacteria with probiotic potential, which have been studied to evaluate their beneficial effects on health [12,13]. The microorganisms used as probiotics are mostly obtained from the intestinal microbiota of humans and animals and are used in commercial products such as fermented milk or lyophilized products administered orally [14,15]. Probiotic bacteria mainly belong to the genus of lactic acid bacteria (LAB) such as *Lactobacillus* and *Bifidobacterum* [16]. There are different species of *Lactobacillus,* including *L*. *acidophilus*, *L. plantarum*, *L. casei*, *L. paracasei*, *L. johnsonii*, *L. reuteri*, and *L. rhamnosus*, which have been considered strong candidates for use as biological therapy [17,18]. Previous studies with *Lacticaseibacillus* strains report inhibitory effects on the growth of enteric pathogens [19,20]. Another property that has been observed in these probiotic bacteria is the inhibition of the growth of pathogenic bacteria through the secretion of ethanol, fatty acids, hydrogen peroxide, bacteriocins, and microcins, products with significant antimicrobial activity [20,21]. Other reports show that the protective effect of probiotic bacteria against pathogenic microorganisms is due to their ability to induce the secretion of mucins that reinforce the protective activity of the epithelial barrier by favoring tighter junctions in the intestinal epithelium [22,23]. In addition, immunomodulatory effects have been observed regulating the secretion of cytokines that activate phagocytic cells and T lymphocytes [24,25]. Other elements that favor the potential of probiotic bacteria include the physicochemical properties of the cell wall such as hydrophobicity, auto-aggregation, and coaggregation, which together with the secretion of short-chain fatty acids (lactic, acetic, and formic acids) form metabolic products released into cell-free supernatants (CFSs), which regulate pH, a situation that can limit the growth of pathogenic microorganisms [26,27,28]. However, to survive, pathogenic bacteria express components that allow them to tolerate adverse conditions such as acidic environments, a lack of nutrients, and the effect of antimicrobials [28,29]. Biofilms form an organized polymeric extracellular structure made up of exopolysaccharides, nucleic acids, and proteins. These allow the bacteria that produce them to adhere to biotic and/or abiotic surfaces, contributing to the colonization of the microorganism while protecting the bacteria from adverse factors such as antimicrobials and even interfering with immune defense mechanisms, which favors the development of infection in the host [29]. Although there are a large number of probiotic bacteria that have been commercialized, many of them have not been evaluated for their ability to interfere with the development of pathogenic bacteria that cause disease in their host. This occurs in such a way that it is necessary to identify in probiotic bacteria for commercial use the properties that they possess to interfere with and/or counteract the virulence mechanisms of pathogenic bacteria. Currently, not all species of probiotic bacteria have been studied in relation to the mechanisms they have to interfere with regarding the growth of pathogenic microorganisms. *Lacticaseibacillus* is a member of the LAB; although it is used as a commercial probiotic, so far there have been no reports to indicate that the properties it possesses inhibit the growth of or eliminate pathogenic bacteria that cause intestinal disease, such as diarrheagenic *E. coli*. In the absence of information about it, the aim of the present study was to investigate the coaggregation ability of this *Lacticaseibacillus* strain and if its CSF has properties such as anti-biofilm activity, an antimicrobial effect, and neutralizing effect on toxins produced by EAEC and EHEC DEC strains. 

## 2. Materials and Methods

### 2.1. Bacterial Strains and Culture Conditions

The *Lactobacillus casei* strain IMAU60214 is an isolate of a fermented dairy product, kindly donated for the study by the research group of Dr. Cruz-Guerrero of Universidad Autónoma Metropolitana de México [30]. The strain was grown in MRS medium (De Manosa, Rogosa, Sharpe) and broth (BDL, Franklin Lakes, NJ, USA) at 37 °C for 18 to 24 h. The pathogenic strains of the DEC group used were EAEC 49766 (a clinical isolate of diarrhea) and EHEC or EDL933 ATCC 700927 as well as a control strain for the experimental tests with *Escherichia coli* ATCC 25922 [31]. These strains were cultivated in Luria–Bertani broth (LB) medium previously prepared according to a certain formulation (5.0 g/Lt sodium chloride, yeast extract, and 10.0 g/Lt casein peptone at pH 7.2) at 37 °C, overnight without agitation. On the test day, the bacteria from the obtained culture were centrifuged at 5000 rpm for 5 min at room temperature. The bacterial cell concentrate was washed three times with physiological saline solution (pH = 7.2) and adjusted to a final concentration of 1 × 10^9^ CFU/mL.

### 2.2. Coaggregation Assay

The coaggregation ability of *Lactobacillus casei* IMAU60214 was tested as reported by Handley et al. [32], with slight modifications. EAEC 49766, EDL933, or ATCC 700927 strains and the *E. coli* test control strain ATCC 25922 were coaggregation partners. Bacterial cultures of the pathogens and of the probiotic *L. casei* IMAU60214 were obtained as indicated in Section 2.1. Subsequently, suspensions of bacterial cells in phosphate saline solution (PBS) pH = 7.2 were prepared in equal volumes of 4 mL and adjusted to a concentration of 1 × 10^9^ CFU/mL. For the coaggregation assays, 2 mL of the *Lactobacillus* and pathogens were mixed in pairs and vortexed for 30 s. The preparations were then incubated at 37 °C, alongside each individual strain of the bacterial cells used as the test control. In addition, the formation of coaggregates with the pathogenic strains was monitored by microscopic visualization in the immersion objective magnification (100×) of each specific pathogenic strain after Gram staining. Immediately, the optical density (OD) was monitored in the mixture (OD mix), that is, of the *Lactobacillus* and the specific strain of the specific pathogen, and in the control tubes containing the *Lactobacillus* strain (OD of the *Lactobacillus*) as the specific pathogen (OD-specific pathogen). Absorbance (ABS) readings were read at intervals of every 15 min up to 120 min using a GeneQuant Pro spectrophotometer (Amerschan Biosciences, Helsinki, Finland) at a wavelength of 600 nm. The percentage of coaggregation was calculated according to the following formula:

% Coaggregation = [1 − OD600 nm mix/control + OD 600 nm (mix) + OD 600 nm specific pathogen/2] × 100

This formula was applied for each incubation interval tested (T1 15 min, T2 30 min, T3 45 min, T4 60 min, T5 90 min, and T6 120 min). Each strain and/or condition was developed in three independent experiments, with each one using duplicate biological samples. 

### 2.3. Preparation of Cell-Free Supernatant (CSF) of L. casei IMAU60214

The *Lactobacillus* strain was grown in MRS broth medium (BDL, Franklin Lakes, NJ, USA) at 37 °C without shaking for 48 h. Immediately, the *Lactobacillus* suspension was centrifuged at 5000 rpm for 5 min and the CSF was collected in sterile tubes. The acid condition of the CSF was adjusted to pH = 6.5 with 1N NaOH. Afterward, the CSF was filtered using 0.22 membranes (Millipore, Burlington, Middlesex, MA, USA). The CFS was used fresh, and its biological activity was tested at different CSF concentrations (10%, 20%, 40%, 60%, and 80%) prepared in stock of volume to volume (*v*/*v*) in MRS broth medium (BDL, Franklin Lakes, NJ, USA). 

### 2.4. Evaluation of the Antimicrobial Activity of CSF against the Diarrheagenic E. coli (DEC) Strains

The antimicrobial activity of CSF from *L. casei* IMAU60214 against diarrheagenic *E. coli* strains EAEC and EHEC was tested by the diffusion plating method described by Davoodabadi et al. [33], with slight modifications. The DEC and *E. coli* ATCC 25922 strains (as the internal positive control) were grown in LB at 37 °C overnight without shaking. The strains were adjusted to a 0.5 McFarland standard tube (1 × 10^8^ CFU/mL), an inoculum of 200 µL of each pathogenic strain was distributed with a cotton applicator moistened with physiological saline solution on Mueller–Hinton agar plates (Difco Agar, Franklin Lakes, NJ, USA), and perforations were made with 1 mL micropipette tips to generate 7 mm-diameter wells. Then, the wells were filled with 100 µL of the CSF concentrations (10 to 80% (*v*/*v*)) prepared in MRS broth medium. The plates were kept for 10 min at room temperature to allow diffusion into the agar; then, they were incubated for 24 h at 35 °C. The activity of each CFS concentration was evaluated by measuring the bacterial radial inhibition zones for each of the DEC strains and the control strain (and/or internal positive control) of *E. coli* ATCC 25922. In addition, a negative control of the MRS broth medium (without adding CFS) was included in the experimental assays, which were performed in duplicate for each of the different CSF concentrations, in a total of three independent experiments.

### 2.5. Biofilm Formation of Diarrheagenic Strains (DEC)

Biofilms of the two DEC strains, EAEC 49766 and EHEC (or EDL933, ATCC 700927), and control strain *E. coli* ATCC 25922 were prepared by adjusting their optical density to McFarland tube 1 (3 × 10^8^ CFU/mL). Then, 50 µL of the adjusted suspension was diluted with 950 µL of LB culture medium and deposited in 24-well polystyrene plates (Costar, (Costar^®^, Corning^®^ Thermo Fisher Scientific, Waltham, MA, USA) prepared in triplicate series of wells. Subsequently, the plates were incubated for 48 h at 37 °C. Afterward, each well was washed six times with phosphate saline pH = 7.2 to remove the suspended bacteria, and the adhered bacteria were fixed with 1 mL methanol (Merck, Darmstadt, Germany) for 15 min at room temperature. The formation of biofilms formed was then quantified and the plates were stained with 400 µL per well of 2% (*v*/*v*) crystal violet solution for 20 min at room temperature. Excess staining was removed by washing with Milli-Q water, and after removing residual fluid, the plates were dried at room temperature. The stain was released by the addition of a 33% (*v*/*v*) glacial acetic acid destaining solution, and ABS at 570 nm was read in a microplate reader (Multiskan, Termolabsystems, Vantaa, Finland). Each assay was carried out in biological triplicates, in a total of three independent experiments.

### 2.6. Evaluation of the Anti-Biofilm Formation Activity of the CSF of L. casei IMAU60214 against the DEC Diarrheagenic Strain

The anti-biofilm formation potential of CSF was tested, considering several mechanisms of action: the inhibition of biofilm formation, the dispersion of pre-formed biofilms, and the effect of catalase and proteinase K proteolytic enzymatic treatment (1 mg/mL) on the biological activity of the CSF of *L. casei* IMAU60214.

#### 2.6.1. Effect of CSF from *L. casei* IMAU60214 on the Biofilm Formation of the DEC Strains

To determine the influence of the CSF of *L. casei* IMAU60214 on the formation of biofilms of the DEC EAEC 49766 and EHEC strains (or EDL933, ATCC 700927) and the control of *E. coli* ATCC 25922, the bacterial suspensions of these strains were adjusted to the optical density of McFarland tube 1 (3 × 10^8^ CFU/mL). Then, 50 µL of the adjusted suspension of each strain was diluted with 950 µL of LB culture medium and deposited in 24-well polystyrene plates (Costar^®^, Corning^®^ Thermo Fisher Scientific, Waltham, MA, USA) in the presence or absence of different concentrations (10–80%) of *L. casei* IMAU60214 CSF in triplicates of biological samples for each of the CSF concentrations [ref]. The cultures were cultivated at 37 °C for 48 h, and after incubation, the development of biofilm formation as well as its quantification was performed as described in Section 2.5. The effect of *L. casei* IMAU60214 CSF on biofilm formation was measured by comparing the absorbance readings at 570 nm from the CSF-treated wells versus the untreated control wells. In addition, the percentage reduction by the CSF of *L. casei* IMAU60214 was calculated according to the following formula:

Percentage reduction in biofilms = 1 − average ABS 570 nm of biological samples/average of the ABS 570 nm control × 100.

Each assay was performed in triplicate on the biological samples, in a total of three independent experiments. In addition, the effect of the presence of the CSF of *L. casei* IMAU60214 on the formation of biofilms of diarrheagenic strains was analyzed on the dry surface of 24-well polystyrene plates (Costar^®^, Corning^®^, Thermo Fisher Scientific, Waltham, MA, USA) in biological samples treated and untreated with the CSF as observed in phase contrast microscopy on a Nikon microscope, using a 20× dry objective (NA075).

#### 2.6.2. Effect of the CSF of *L. casei* IMAU60214 on the Dispersion of Biofilms of the DEC Strains 

Experimental biofilm dispersion assays of the DEC strains’ pre-formed (mature) biofilms were prepared in 24-well plates (Costar^®^, Corning^®^, Thermo Fisher Scientific, Waltham, MA, USA) as described in Section 2.5. Briefly, pre-formed biofilms from each DEC strain and the control *E. coli* ATCC 25922 were treated with varying concentrations (10 to 80%) of *L. casei* IMAU60214 CSF in parallel with assaying untreated adherent bacterial cell wells and the blank well control. The 24-well plates, under these conditions, were incubated at 37 °C for 24 h. The dispersion or eradication of biofilms was calculated according to the following formula:

Eradication or dispersion of biofilms = 1 − mean ABS 570 nm of biological samples/mean of the ABS 570 nm control × 100

#### 2.6.3. Evaluation of the Anti-Biofilm Activity and Biofilm Dispersion of the DEC Strains after Proteolytic Enzymatic Treatment with the Catalase and Proteinase K of the CSF of *L. casei* IMAU60214

The effects of proteolytic enzymatic treatment with catalase and proteinase K on the biological activity of the CSF of *L. casei* IMAU60214 were investigated in relation to the formation and dispersal of the biofilms of the DEC strains. A stock solution of catalase (Sigma, Aldrich St. Louis, Missouri, USA ) was prepared at 1 mg/mL in 0.2 M PBS pH = 7.0, while a stock solution of proteinase K (Thermo Fisher Scientific, Waltham, MA, USA) at the same concentration was prepared in 30 nM PH tris buffer solution equal to 7.5. Subsequently, the CSF preparations were incubated (1:1 *v*/*v*) with the enzyme solutions for two hours at 37 °C. After these treatments, the formation as well as the dispersion of DEC biofilms were carried out as described in Section 2.6.1 and Section 2.6.2. CSF negative controls (without enzymatic treatment) were included in the experimental assays carried out on the biological samples in triplicate for a total of three independent experiments. 

### 2.7. Culture of the HT-29 Human Intestinal Epithelium Cells

Primary-colon-tumor-derived adenocarcinoma HT-29 cells (ATCC HT-38) were routinely grown in DMEM (Dulbecco’s modified Eagle medium) with 10% (*v*/*v*) fetal bovine serum (FBS) without antibiotic supplementation in 25 cm^2^ cell culture bottles (Costar, Corning) at 37 °C in a humid atmosphere of 5% CO2. Monolayers of the HT-29 cells were prepared in 96-well polystyrene cell culture plates (Costar^®^, Corning^®^, Thermo Fisher Scientific, Waltham, MA, USA) and grown to 90% confluence. Subsequently, the medium was replaced with fresh 10% DMEM/SFB medium 24 h before the co-cultivation study of HT-29 human epithelial cells infected with the diarrheagenic *E. coli* strains and the control *E. coli* ATCC 25922. 

### 2.8. Infection Assay of the HT-29 Epithelial Cells with the DEC Strains

Confluent monolayers of HT-29 cells were infected with *E. diarrhoeagenica* strains DEC, EAEC 49766, and EHEC (or EDL933, ATCC 700927) and *E. coli* strain ATCC 25922 at a multiplicity of infection (MOI) of 1:10 bacteria versus cells eukaryotes. The bacteria in co-culture with the HT-29 cells were incubated at 37 °C in a humid atmosphere of 5% CO_2_ for 4 h. After the co-culture period, the bacteria were removed from the monolayers by washing with DMEM (Gibco^TM^, Thermo Fischer Scientific, Waltham, MA, USA) and the cells were processed to examine the cytotoxic effect produced by these bacteria by phase contrast microscopy using a 20× objective lens with image capture examining the selected fields. Moreover, the post-infection cellular cytotoxicity analysis used the reduction with MTT (3-[4,5-dimethyl-2-yl]-2,5 diphenyl tretrazolium bromide). 

### 2.9. Cellular Cytotoxicity Assay in HT-29 the Human Epithelial Cells Post Infection with the Escherichia coli DEC Strain 

Cellular cytotoxicity was assayed by the MTT reduction method described by Denizot et al. [34] with slight modification. Briefly, after post-infection with the DEC strains, the medium was removed, and the cells were washed with DMEM containing 100 µg/mL gentamicin (Gibco^TM^, Thermo Fisher Scientific, Waltham, MA, USA). Subsequently, the HT-29 cells were washed with a PBS pH = 7.0 solution and incubated at 37 °C for 4 h with an MTT solution (0.5 mg/mL). At the end of the incubation period, the MTT solution was removed and dimethylsulfoxide (DMSO, Merck) was added. The MTT reduction was read in a microplate reader (Multiskan, Termolabsystems Fildandia) at a wavelength of 570 nm. Cellular cytotoxicity was expressed in percentage values, in comparison with the negative controls (uninfected cells). The assays were developed in duplicate biological samples for a total of three independent experiments. 

### 2.10. Evaluation of the Effect of the CSF from L. casei IMAU60214 on the Co-Culture of HT-29 Human Epithelial Cells Infected with the Bacterial Cells of the DEC 49766 EHEC and EDL933 Strains

In order to understand the impact of the CSF of *L. casei* IMAU60214 on the cell damage induced by the infection of human epithelial cells HT-29 in response to the challenge of DEC strains and the control of *E. coli* ATCC 25922, the bacteria were added in a multiplicity of infection (MOI) ratio of 1:10 to HT-29 cell monolayers seeded in 96-well plates in co-culture of different CSF concentrations (10–80%), which were incubated at 37 °C in a humid atmosphere of 5% CO_2_ for 4 h. After the incubation period, the plates were washed three times with warm PBS solution and the viability for the different experimental conditions mentioned above including the untreated HT-29 epithelial cell controls was determined by the MTT reduction method as indicated in Section 2.9. Alternatively, morphological changes observed on the monolayers of HT-29 epithelial cells post infection with the DEC strains and control *E. coli* ATCC 25922 were photographed by phase contrast microscopy under a Nikon microscope using a 20× dry objective (NA075). Each assay was carried out in biological triplicates in a total of three independent experiments.

### 2.11. Statistical Analysis

The results are expressed as the mean ± standard deviation (SD) of three independent experiments. Statistical analysis was performed using the GraphPad Prism version 6 program. The data obtained were subjected to an analysis using one-way (ANOVA), with Turkey’s post hoc test by using GraphPad Prism (San Diego, CA, USA) and a statistically significant difference was considered with *p* < 0.05.

## 3. Results

### 3.1. Coaggregation of L. casei IMAU60214 with the DEC Strains 

**Figure 1:**
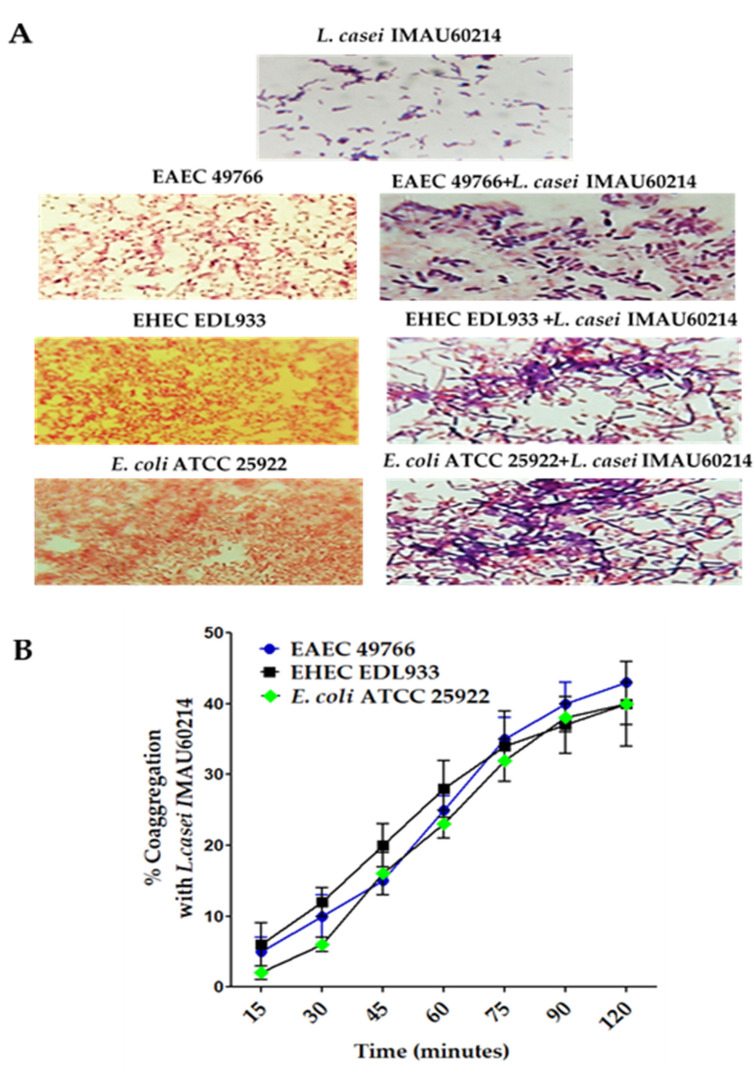
Coaggregation activity of *L. casei* IMAU60214 with three pathogenic strains (two DEC strains EHEC, EAEC, and the control *E. coli* ATCC 25922). (**A**) The formation of bacterial coaggregates of the study strains were observed by Gram-staining and visualized with the immersion objective using a light microscope Nikon. The magnification of the picture is 1000× (**B**) Coaggregation percentages with the *E. coli* strains after 120 min of interaction at 37 °C. The values represent the mean ± standard deviation of three independent biological assays. The magnification of these picture is 100×.

According to the physicochemical characteristics of coaggregation in Figure 1, it was observed that *L. casei* IMAU60214 has an evident interaction with the diarrheagenic *E. coli* strains evaluated, forming visible coaggregates (Figure 1A). It was also observed that after 120 min of incubation with *L. casei* IMAU60214 (Figure 1B), the values of the coaggregation capacity of the *Lactobacillus* with each of the three *E. coli* strains tested increased with time, observing intervals of 1% to 40% with respect to time, specifically the coaggregation capacity with DEC strains EAEC 49766 (32 ± 5%), EHEC DL933 (40 ± 2%), and *E. coli* ATCC 25922. 

### 3.2. Antimicrobial Activity of the Cell-Free Supernatant (CSF) of L. casei IMAU60214 against Diarrheagenic E. coli 

**Figure 2:**
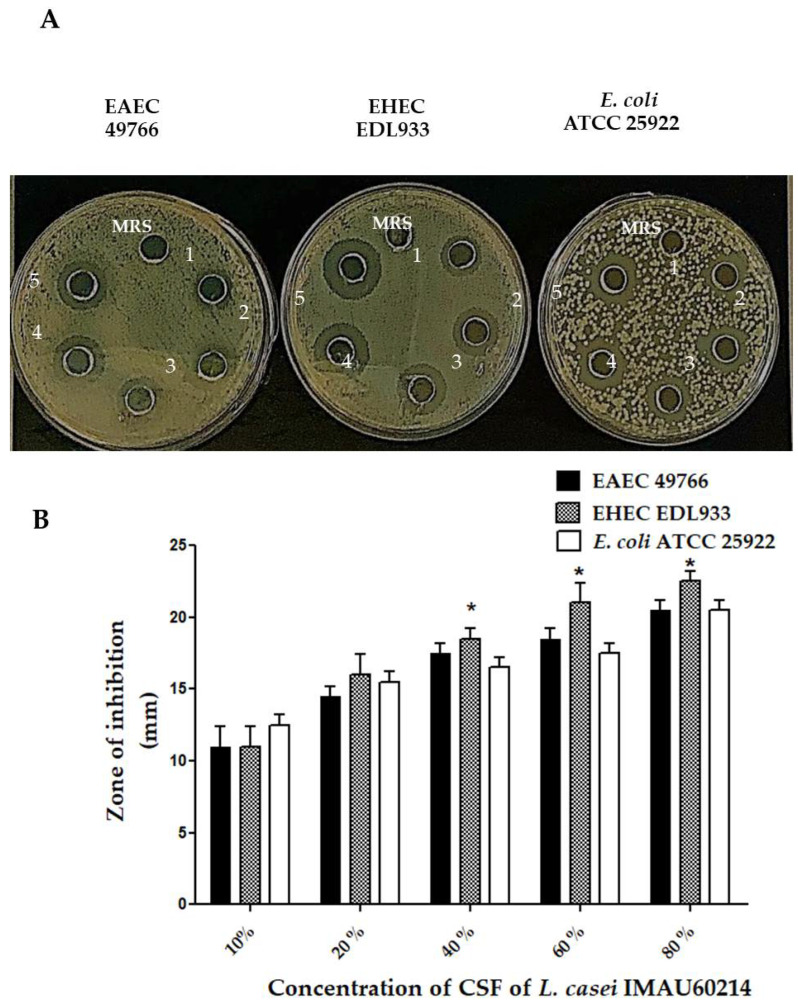
Antimicrobial activity of (CSF) from *L. casei* IMAU60214 against the DEC strains. (**A**) Representative images of growth inhibition zones of the pathogenic strains at different concentrations of CSF pH = 6.5 (10–80%) deposited in wells (1–5) and the negative control (MRS medium alone) on Mueller–Hinton agar plates after incubation for 24 h at 35 °C. (**B**) Measurement of zones of inhibition (mm) where the values represent the mean ± standard deviation of three independent biological assays for each pathogenic strain. * significant difference among the concentrations of CSF (*p* < 0.05).

The agar diffusion assays used to analyze the antimicrobial activity of the CSF from *L. casei* IMAU60214 showed growth inhibition against EAEC and EHEC strains and the control strain *E. coli* ATCC 25922 Figure 2. The same assay without adding CSF with only MRS medium showed no growth inhibition after 24 h of incubation at 35 °C (Figure 2A). The inhibition halos obtained ranged from 10 ± 2 mm to 20 ± 2.5 mm in diameter among the DEC strains (Figure 2B). In addition, the intensity of inhibition was observed to be dependent on the CSF concentration (10–80%).

### 3.3. Inhibitory Effect of the CFS from L. casei IMAU60214 on the Biofilm Formation of the DEC Strains 

**Figure 3:**
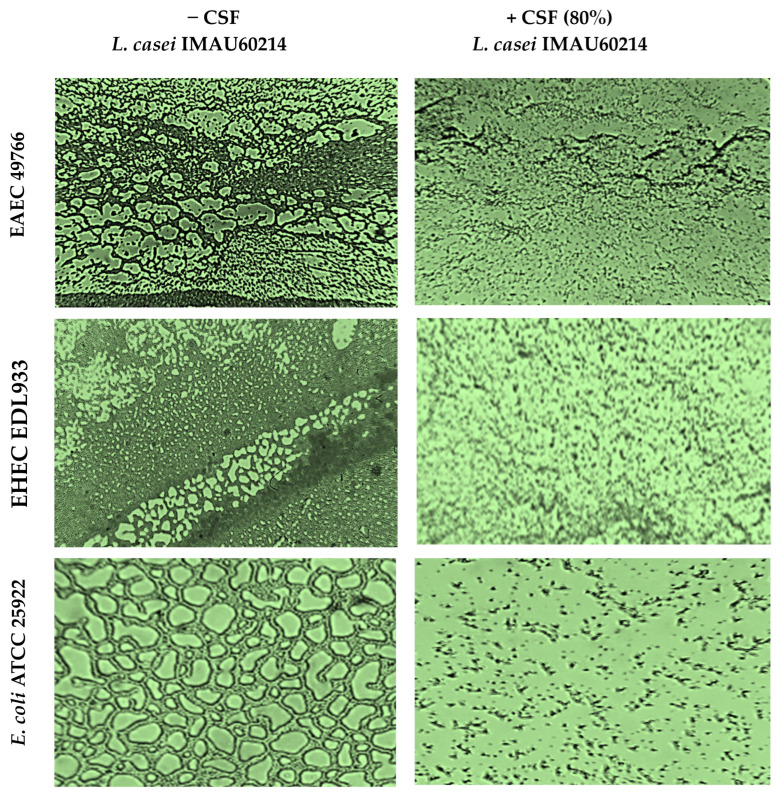
Structural examination of the effect of the CSF from *L. casei* IMAU60214 on the formation of mature biofilms of the DEC strains on the abiotic surface. Biofilm formation in the presence of 80% CSF concentration and in its absence was analyzed using a Nikon inverted phase contrast microscope with a dry objective, the magnification of these picture is 1000×. The images shown are representative of three independent biological assays.

The formation of biofilms developed on abiotic surfaces and the effect of the CSF of *L. casei* IMAU60214 were analyzed by phase contrast microscopy as shown in Figure 3. Structural observation of the biofilms from the DEC and control *E. coli* ATCC 25922 strains in the absence of *L. casei* IMAU60214 CSF treatment showed an organized architecture of mature biofilms in both the DEC and control *E. coli* ATCC 25922 strains. Among the features associated with mature biofilms, fungal formations, microcolonies, monolayers, and multilayers were observed on the abiotic surface; each DEC strain (EAEC 49766 and EHEC EDL933) showed its own structural pattern in the formation of mature biofilms. The same assay but treated with *L. casei* CSF IMAU60214 at 80% (*v*/*v*) showed a reduction in the formation of mature biofilms with the loss of their organization.

### 3.4. Effect of the CFS from L. casei IMAU60214 on the Formation and Dispersion of DEC Biofilms 

**Figure 4:**
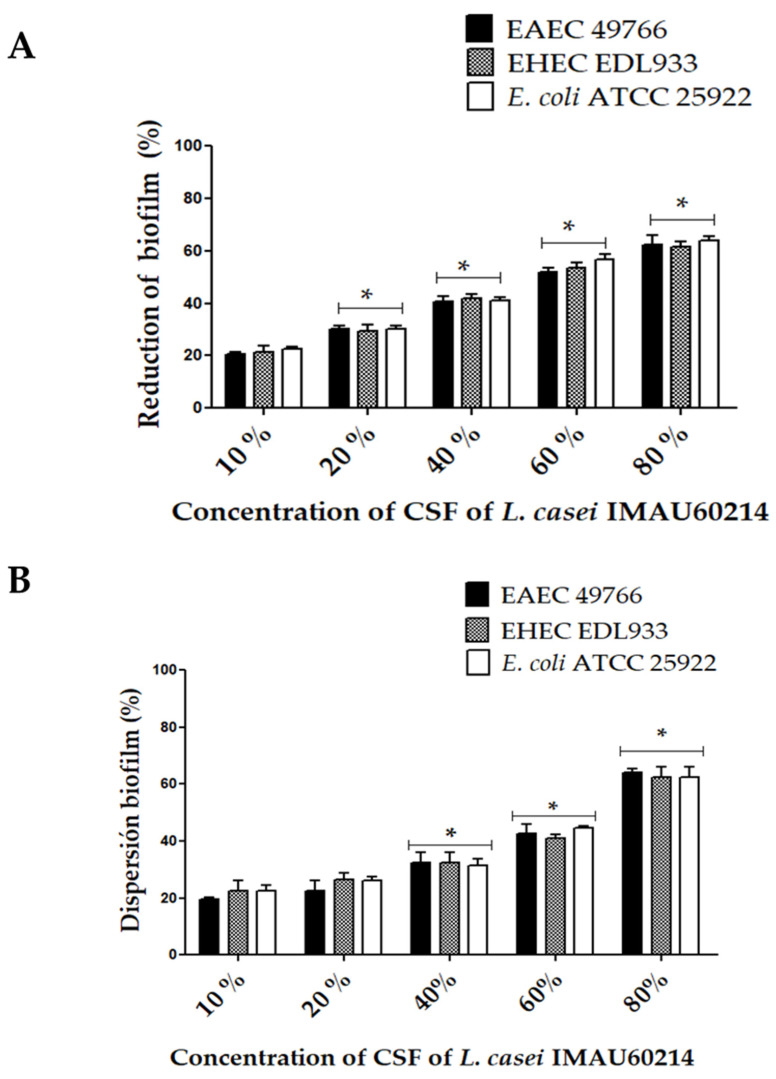
Impact of CSF from *L. casei* IMAU60214 on the biofilm formation and dispersion of the DEC strains on the abiotic surface. (**A**) Percentage of biofilm formation in the presence of different concentrations of CSF (10–80%) quantified by the crystal violet method. (**B**) Percentage of mature biofilm dispersion after 24 h of CSF treatment (10–80%). The values represent the mean ± standard deviation of three independent biological assays for each of the pathogenic strains. * significant differences among the concentrations of CSF (*p* < 0.05).

The effect of the CSF from *L. casei* IMAU60214 on the formation and quantitative dispersion of biofilms of DEC strains are shown in Figure 4. The presence of CSF significantly reduced the formation of bacterial cell biofilms with respect to the control DEC strains and untreated *E. coli* ATCC 25922 (Figure 4A). Moreover, in this assay, the effect was dependent on the concentration of CSF; the quantitative analysis of the reduction effect with CSF for each strain evaluated at high concentrations of CSF (40–80%) was 52 ± 5% in EAEC 49766, 40 ± 2% in EHEC DL933, and 35 ± 5% in *E. coli* ATCC 25922. The assay further showed that CSF treatment causes the dispersion of pre-formed (mature) biofilms of DEC strains on the abiotic surface after 24 h (Figure 4B). Concentrations of 40%, 60%, and 80% *v*/*v* of CFS dispersed DEC biofilms of 35 ± 3%, 50 ± 5%, and 62 ± 3%, respectively. This dispersion property of the CSF is concentration dependent. Furthermore, it was observed that this dispersion is similar between the DEC strains and the control strain. 

### 3.5. Effect of CSF Pretreated with Catalase and Proteinase K on the Biofilm Formation and Dispersion Activity of the DEC Strains 

**Figure 5:**
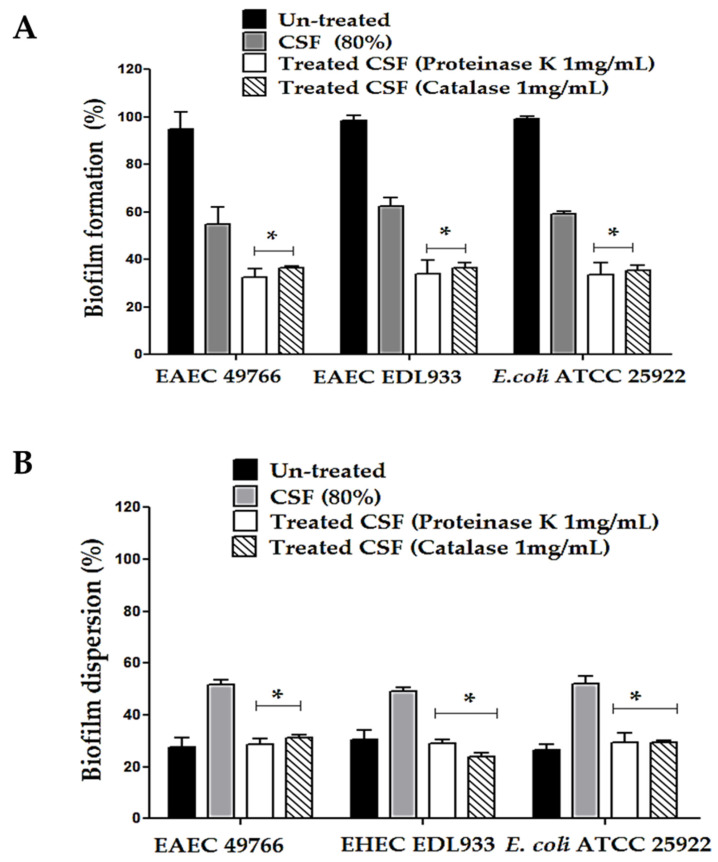
Effect of enzymatic pre-treatment with proteinase K and catalase (1 mg/mL) from the CSF of *L. casei* IMAU60214 on the formation and dispersion of biofilms of the DEC strains as well as the control *E. coli* ATCC 25922 strains on the abiotic surface. (**A**) Biofilm formation; (**B**) biofilm dispersion. The values represent the mean ± standard deviation of three independent biological assays for each of the pathogenic strains. * significant difference among the treatments (*p* < 0.05).

In the search for metabolites involved in the functional activities of CSF from *L. casei* IMAU60214 against DEC strains, the effect of biofilm formation and dispersion was analyzed in the presence of oxidative components such as hydrogen peroxide and in the possible presence of proteins with bacteriocin function (Figure 5). Biofilm formation was significantly abated between 35 ± 3 and 30 ± 2% in the presence of the enzyme catalase compared to the untreated CSF controls in all of the study strains (Figure 5B). Similar data were observed when the CSF was pre-treated with proteinase K at a concentration of 1 mg/mL in both the biofilm formation and the dispersion of the DEC strains (Figure 5B).

### 3.6. Effect of CSF Pretreated with Catalase and Proteinase K on the Biofilm Formation and Dispersion Activity of the DEC Strains 

In the search for metabolites involved in the functional activities of CSF from *L. casei* IMAU60214 against the DEC strains, the effect of biofilm formation and dispersion was analyzed in the presence of oxidative components such as hydrogen peroxide and in the possible presence of proteins with bacteriocin function as shown in Figure 5. Biofilm formation was significantly abated between 35 ± 3 and 30 ± 2% in the presence of the enzyme catalase compared to the untreated CSF controls in all study strains (Figure 5B). Similar data were observed when the CSF was pre-treated with proteinase K at a concentration of 1 mg/mL in both the biofilm formation and the dispersion of the DEC strains (Figure 5B). 

### 3.7. Effect of the CSF from L. casei IMAU60214 in an In Vitro Co-Culture with Human HT-29 Epithelial Cells Infected with the DEC Strains 

**Figure 6:**
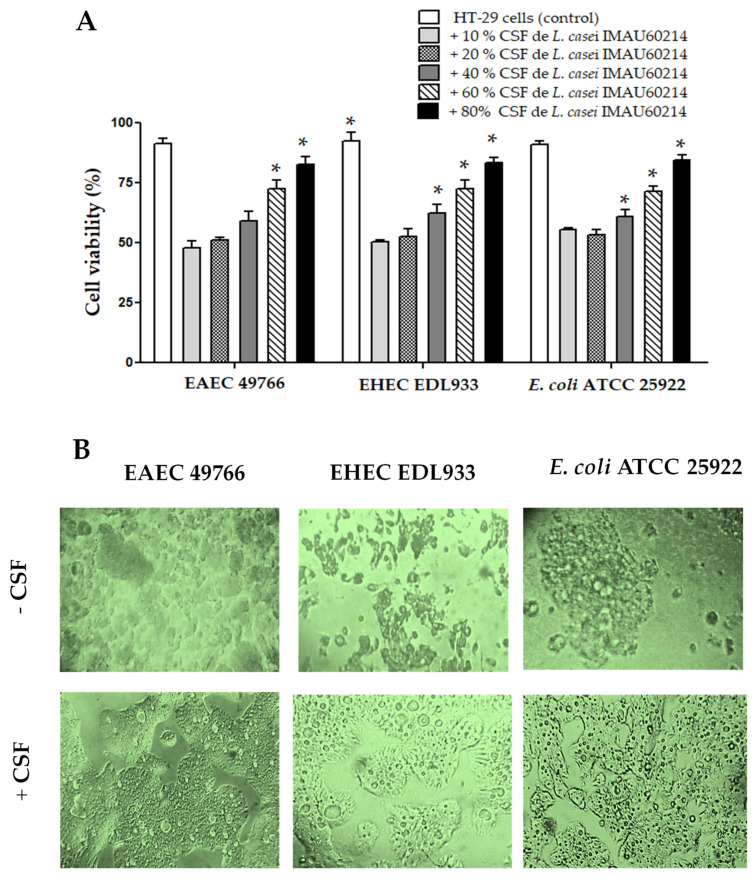
Effect of CSF co-incubation from *L. casei* IMAU60214 on the cytotoxic damage induced with the DEC strains on HT-29 human epithelial cells. (**A**) Quantification of cell damage by MTT reduction. (**B**) Representative images of the structural analysis by visualization in phase contrast microscopy using an inverted microscope with a 20× dry objective in the absence of CSF and with CSF (80%). The magnification of these picture is 400×. The values represent the mean ± standard deviation of three independent bioassays for each of the pathogenic strains. * significant difference among the treatments (*p* > 0.05).

The influence of CSF on cell damage and/or cytotoxicity induced by in vitro infection with DEC strains on HT-29 epithelial cells is shown in Figure 6. The cytotoxicity data quantified by MTT reduction exhibit a positive impact, with a significant dependence on the concentration of CSF present in the co-culture. Concentrations of 40% to 60% (*v*/*v*) of (CSF) maintain viability at levels similar to uninfected cells (Figure 6A) 

In contrast, the HT-29 cells infected with each of the DEC strains exhibit lower viability (30 ± 5% to 50 ± 3%); that is, more than half of the HT-29 cells infected with diarrheagenic *E. coli* show cell cytotoxicity effects, supported by the reduction in metabolic activity quantified by MTT reduction. It is noteworthy that the EAEC and EHEC strains produce greater damage compared to that caused by the control strain of *E. coli* ATCC 25922. Similarly, when analyzing the morphological changes in uninfected cells and cells infected with diarrheagenic *E. coli*, we found distinct morphological changes associated with cell damage (Figure 6B). 

The observations made by phase contrast microscopy show the loss of the integrity of the monolayer, a reduction in cell size, and increased vacuolization of the cells compared to that observed in the uninfected cells. On the other hand, the structural monolayer of the cells co-cultured with CFS and infected with the diarrheagenic *E. coli* strains closely resembles the cell monolayers of the negative control (uninfected cells). Analysis of cytotoxicity percentages of HT-29 human epithelial cells co-cultured with CFS infected with DEC strains shows a significant reduction in the concentration-dependent cytotoxic effect of *L. casei* CSF IMAU60214.

## 4. Discussion 

In recent decades, the extensive use of antibiotics against pathogenic bacteria such as *Klebsiella pneumoniae*, *Staphylococcus aureus*, *Streptococcus pneumoniae*, and *Salmonella* spp., among other microorganisms has increased antibiotic resistance, limiting their treatment options [35,36]. Previous studies have demonstrated that the Enterobacteriaceae family includes genetic variants resistant to carbapenems [37]. In this context, recent studies have focused on using this family as an alternative strategy probiotic strain and/or its metabolic bioproducts released in cell-free supernatants (CSFs) of *Lactobacillus* spp. which showed activity against carbapenem-resistant strains of the Enterobacteriaceae family [38]. In the intestinal environment, probiotic strains can exert their beneficial potential through their properties of coaggregation, competitiveness, pathogen exclusion, and adhesion prevention on the intestinal mucosa, among other parts of the human body [39]. The most promising scenario of probiotic potential has been established through in vitro and in vivo studies that have analyzed the antimicrobial activity attributable to metabolic bioproducts [40,41]. In the present study, the potential antimicrobial activity of *L. casei* CFS IMAU60214 obtained from a dairy isolate was evaluated. The evaluation was performed on an important group of enteric pathogenic bacteria, mainly Escherichia coli pathotypes causing diarrhea, such as EAEC, and other disorders, such as hemolytic uremic syndrome, caused by EHEC [42,43]. An interesting observation was the ability of *L. casei* IMAU60214 to coaggregate against two DEC strains and the reference strain *E. coli* ATCC 25922. Coaggregation ability is a physicochemical property of the bacterial cell wall, which favors cell–cell interaction that conditions the formation of a barrier that interferes with the adherence of pathogenic bacteria to the intestinal mucosa and prevents them from colonizing the intestinal mucosa [44,45]. Previous reports refer to coaggregation activities between strains of *L. paracasei* and *Lactobacillus rhamnosus* with pathogenic *E. coli* [46,47]. However, other investigations report the existence of *Lactobacillus* strains with limited ability to coaggregate with pathogens, suggesting that there is variability among strains and species of probiotic bacteria against pathogenic microorganisms [48,49]. The results of the present study concerning the coaggregation property of viable *L. casei* IMAU60214 suggest that this probiotic strain is a good candidate for controlling infection caused by diarrheagenic *E. coli*. Furthermore, the coaggregation activity (pathogen–probiotic strain), in addition to interfering with the adherence and colonization of the pathogen, can maintain a microenvironment that increases the efficacy of the antimicrobial action of the bioproducts secreted by the probiotic bacteria. In this regard, it has been hypothesized that properties such as coaggregation and antimicrobial effects could contribute to the decrease in pathogen load by competitive exclusion mechanisms during infections [50,51]. In this study, we observed that the CSF from *L. casei* IMAU60214 had an antimicrobial effect on the study strains EAEC, EHEC, and *E. coli* ATCC 25922. It is important to note that antimicrobial activity continues to be one of the main criteria for the selection of probiotic strains. Biofilm formation is an important property expressed by some pathogenic bacteria, so having innocuous products that interfere with the formation of biofilms of pathogenic microorganisms would contribute to their elimination. Biofilms, in addition to contributing to the adherence of bacteria, protect them against the action of the immune response and the action of antimicrobials [52,53]. When evaluating the CSF activity of *L. casei* IMAU60214, it was observed that it reduces and disintegrates biofilms of DEC strains. The DEC strains used in the study are excellent biofilm producers, a relevant aspect since not all pathogenic microorganisms present this activity [54,55]. The impact of CSF on the formation and dispersion of biofilms against these pathogenic DEC strains provides evidence that *L. casei* IMAU60214 is an excellent candidate to be used as a useful probiotic bacterium for the control and treatment of infections related to infection by *E. coli* strains that cause diarrhea and other more severe conditions such as hemolytic uremic syndrome (HUS) [56]. The analysis of the components potentially involved in the mechanisms of action of CSF revealed that both the anti-formation and biofilm dispersion activities of the DEC strains were reduced by treatment with proteinase K and catalase individually. These results indicate that, most probably, the components responsible for these activities are proteins that need to be identified and oxidizing compounds, particularly hydrogen peroxide. In previous works, it has been reported that metabolites of a protein nature, oxidative components, and organic acids contained in or derived from CSFS can alter the pathogenesis of certain enteric bacteria such as *Salmonella enteritidis* [57]. Structural analysis of the biofilms through phase contrast microscopy imaging provided evidence of the ability of the components released in the CSF of *L. casei* IMAU60214 to inhibit the architectural development of biofilms formed by the DEC strains. It was also observed that the CSF from *L. casei* IMAU60214 exhibits a protective effect on HT-29 epithelial cells infected with the EAEC and EHEC strains, producing toxins such as Pet toxin and Shiga-like toxin (SLT), respectively [58,59,60,61,62]. In this regard, Navarro et al. [63] showed that Pet secreted by strains of the EAEC pathotype induces morphological changes in the mucosa characterized by flattening of the intestinal villi with cell damage to the intestinal mucosa evaluated in an experimental rat ligated loop model. In the same context, SLT from EHEC has cytotoxic capacity, causing cell damage to the human intestinal epithelium [64]. In agreement with the results obtained in the present study, recent reports show that the CSF from *L. casei* causes destruction of the bacterial cell membrane. The results of this work agree with those reported in other investigations that suggest that supernatants derived from probiotic bacteria significantly reduce cytotoxic effects and apoptosis induced by enteric pathogens, such as *Salmonella* Typhimurium [65].

## 5. Conclusions 

It can be concluded that both the coaggregation effect and the CSF activity of *L. casei* strain IMAU60214 make this bacterium a suitable candidate for use as a probiotic with potential to interfere with the activity of some of the pathogenic factors of diarrheagenic *E. coli* strains. The analysis of the literature indicates that the present study is the first to contribute to the characterization of the antagonistic activity of *L. casei* IMAU60214, against DEC strains. However, it presents some limitations. One of these is related to the biofilm formation assay that was only analyzed on abiotic surfaces; it would have been important to demonstrate that this CSF supernatant can destroy biofilm formation under biotic conditions. Another aspect that remains to be evaluated is the total CSF composition of this *L. casei* strain IMAU60214. However, what has been shown is the basis for confirming its impact through extensive research in both in vivo studies with animal models and clinical studies in the future, in which there will be strong reasons to consider it as a therapeutic alternative for infections caused by diarrheagenic *E. coli.*

## Data Availability

The data presented in this study are available upon request from the corresponding author.
